# Injectable nanohydroxyapatite-chitosan-gelatin micro-scaffolds induce regeneration of knee subchondral bone lesions

**DOI:** 10.1038/s41598-017-17025-6

**Published:** 2017-12-01

**Authors:** B. Wang, W. Liu, D. Xing, R. Li, C. Lv, Y. Li, X. Yan, Y. Ke, Y. Xu, Y. Du, J. Lin

**Affiliations:** 1 Arthritis clinic and research center, Peking University People’s Hospital, Beijing, 100044 People’s Republic of China; 2 Department of Biomedical Engineering, School of Medicine, Collaborative Innovation Center for Diagnosis and Treatment of Infectious Diseases, Tsinghua University, Beijing, 100084 People’s Republic of China; 3 Department of Orthopaedics, The Second Hospital of Shanxi Medical University, Taiyuan, Shanxi 030001 People’s Republic of China

## Abstract

Subchondral bone has been identified as an attractive target for KOA. To determine whether a minimally invasive micro-scaffolds could be used to induce regeneration of knee subchondral bone lesions, and to examine the protective effect of subchondral bone regeneration on upper cartilage, a ready-to-use injectable treatment with nanohydroxyapatite-chitosan-gelatin micro-scaffolds (HaCGMs) is proposed. Human-infrapatellar-fat-pad-derived adipose stem cells (IPFP-ASCs) were used as a cellular model to examine the osteo-inductivity and biocompatibility of HaCGMs, which were feasibly obtained with potency for multi-potential differentiations. Furthermore, a subchondral bone lesion model was developed to mimic the necrotic region removing performed by surgeons before sequestrectomy. HaCGMs were injected into the model to induce regeneration of subchondral bone. HaCGMs exhibited desirable swelling ratios, porosity, stiffness, and bioactivity and allowed cellular infiltration. Eight weeks after treatment, assessment via X-ray imaging, micro-CT imaging, and histological analysis revealed that rabbits treated with HaCGMs had better subchondral bone regeneration than those not treated. Interestingly, rabbits in the HaCGM treatment group also exhibited improved reservation of upper cartilage compared to those in other groups, as shown by safranin O-fast green staining. Present study provides an in-depth demonstration of injectable HaCGM-based regenerative therapy, which may provide an attractive alternative strategy for treating KOA.

## Introduction

Knee osteoarthritis (KOA) is the most common disorder and leading cause of disability in the elderly, with the prevalence of symptomatic KOA reaching approximately 8.1% among Chinese adults^1,2^. The features of KOA include not only cartilage damage, synovitis inflammation, and osteophyte formation; but also subchondral bone lesions^3^, such as bone marrow lesion and cyst formation^4^, which are associated with KOA development^5–7^. As the integrity of subchondral bone is essential for upper cartilage functions, pathological changes to subchondral bone can lead to a disrupted joint surface and cartilage damage during KOA development^8^. Thus, subchondral bone has been identified as an attractive target for treatment of KOA^9^.

With the concept of phenotype-based therapy for KOA, effective disease-modifying therapies have mostly been used on cartilage lesions, extensive synovitis and bone marrow lesions individually for structural and symptom modification^10^. In current clinical practice, therapeutic strategies for subchondral bone lesions include antiresorptives, bone-forming agents, antiosteoporotic drugs^9,11^, treadmill^12^, distraction^4^ and subchondroplasty by injection of calcium phosphate^13^. The ultimate goal of these therapies is to realize augmentation of subchondral bone remodelling, which then improve structural mineral density and the integrity of damaged subchondral bone^9^. However, current therapeutic strategies show limited efficiency due to the lack of precision and long term effects, and no regenerative therapies have been proven to be effective for treating existing inactivated subchondral bone lesions. Such as: subchondral bone cysts, as one type of sealed and undynamic subchondral bone lesions, are a common finding in patients with knee osteoarthritis and highly associated with osteoarthritis. There are two proposed theories regarding how cyst formation occurs: the synovial breach theory; the bony contusion theory. Furthermore, subchondral bone cysts in the knee need to be treated because they are associated with an increased risk of knee arthroplasty^14^ and even joint revision^15^. To the best of our knowledge, there is still no specialized treatment for regenerating subchondral bone cysts. Among the salvage procedures, necrotic region removing combined with or without bone graft substitutes maybe a viable option.

To diminish surgical trauma and simplify surgical procedures, injectable bone biomaterials have been widely studied for minimally invasive regenerative therapy. The injectable characteristic of these biomaterials is due to their intrinsic chemical or physical properties. In our previous work, we established 3D micro-scaffolds as injectable cell delivery vehicles for treating challenging regenerative diseases such as critical limb ischemia (CLI)^16^, and intervertebral disc degeneration^17^. In both works, we successfully realized cell-based therapy by greatly attenuating tissue damages. However, clinicians have been looking for a ready-to-use biomaterial; that does not contain growth factors or cells; and can rapidly be tailored for different shapes and sizes^18,19^. Significant efforts have been made in previous decades to propose biomaterials suitable for bone regeneration^20,21^. Among which, biomimetic nanohydroxyapatite/chitosan/gelatin (HaCG) scaffolds were a popular choice owing to their ability to stimulate constituent components of natural bone, guide the regeneration progress, and degrade to nontoxic byproducts^22,23^. Nanohydroxyapatite (HA), the core component of this composite biomaterial, has been shown not only to promote cell adhesion, and support long-term growth, but also to help induce increased cell proliferation and differentiation towards a bone lineage^24,25^. Chitosan possesses inherent physical and biological characteristics that render it useful as a components in bone tissue engineering^20,23,26^; it even has a bactericidal effect^27^. Gelatin, a hydrolysed collagen product with high hydrophilicity, enhances cell adhesion and provides functional groups for chemical crosslinking to form scaffolds^28^. Additionally, all three components are approved by the FDA for clinical use^29^.

In the present study, we innovatively fabricated elastic HaCG micro-scaffolds (HaCGMs) for use as an injectable bone biomaterial for minimally invasive treatment. These injectable micro-scaffolds were designed based on the hypothesis that a favourable microenvironment for cell infiltration, proliferation and nutrient exchange could be provided due to the desirable swelling ratio, porosity, stiffness, osteoinductivity, biocompatibility, and biodegradability properties of these micro-scaffolds. To evaluate the osteoinductivity and biocompatibility of HaCGMs, human-infrapatellar-fat-pad-derived adipose stem cells (IPFP-ASCs), which are multi-potent and obtainable from feasible sources, were used for assessment *in vitro*. We then established a sealed subchondral bone lesion model in rabbits by utilizing the cavity-filling strategy to mimic the actual necrosis removing of subchondral bone cysts performed by surgeons before sequestrectomy. HaCGMs were then injected into the damaged area for treatment to evaluate the osteoinductive and regenerative capacity of the micro-scaffolds and their ability to protect subchondral bone regeneration for upper cartilage maintenance *in vivo*.

This study demonstrates a cavity-filling strategy to establish a sealed subchondral bone lesion animal model to accelerate pathological and clinical intervention research; and provides an attractive alternative biomaterial-assisted therapeutic option for KOA treatment.

## Results

### Experimental design

**Figure 1:**
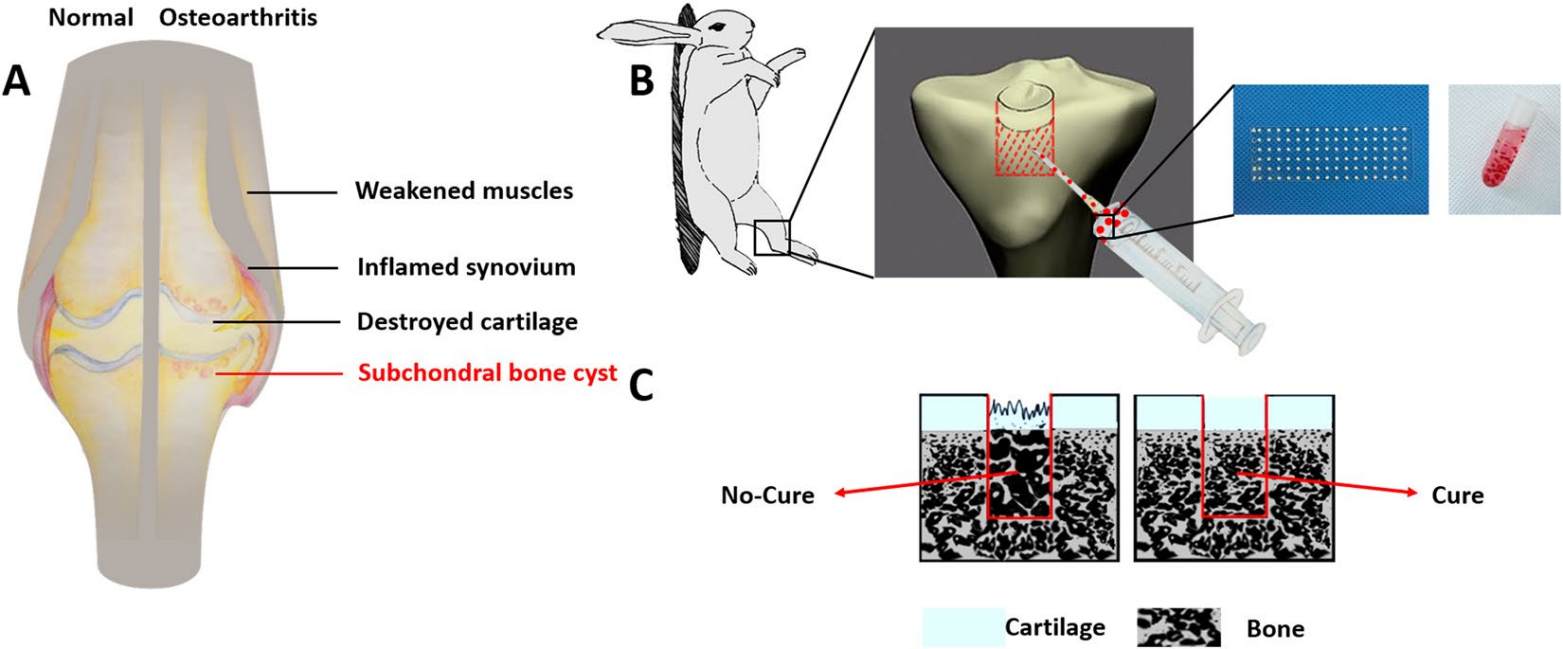
Schematic illustration of overall research design. (**A**) Articular structures that are affected between normal and osteoarthritis tissues, including damaged cartilage, weakened muscles, inflamed synovium, and subchondroal bone cysts. (**B**) Schematic showing the subchondral bone lesion model that was established to mimic the reparative progress using injectable HaCGMs to fill a cavity. HaCGMs stained red solely for visualization purpose were homogeneously suspended in 20% gelatin solution. (**C**) Regeneration of subchondral bone lays the foundation for the upper cartilage. We got permission from Xiuxiu Wang for publication of the image.

The overall research design is shown in Fig. 1. Damaged cartilage, weakened muscle, inflamed synovium, and subchondral bone cysts are found in osteoarthritis development (Fig. 1A). A sealed subchondral bone lesion model was established to mimic the reparative process using injectable HaCGMs to fill a cavity. HaCGMs, stained red solely for visualization purposes, were suspended homogeneously in 20% gelatin solution (Fig. 1B). Regeneration of subchondral bone plays a crucial role in supporting the upper cartilage (Fig. 1C).

### Characterization of HaCGMs

**Figure 2:**
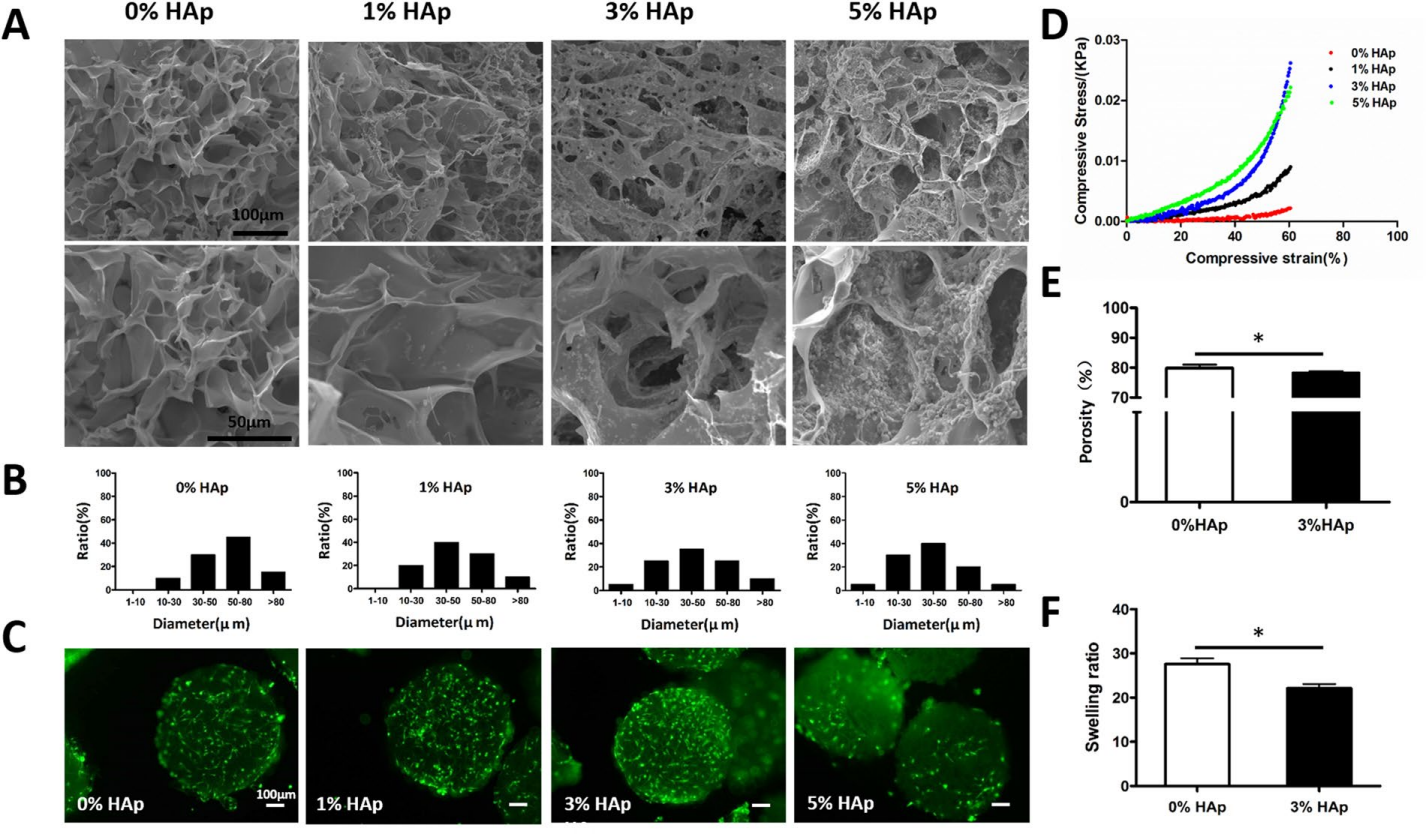
Scanning electron microscopy (SEM) images, pore size statistics and characteristics of HaCGMs: (**A**) SEM images of HaCGMs with HAp (0%, 1%, 3% and 5%) showing interconnected macroporous structures. (**B**) The pore size distribution of HaCGMs with HAp (0%, 1%, 3% and 5%). (**C**) Live/dead staining with Calcein AM/PI showed that IPFP-ASCs seeded in HaCGMs with 3% HAp have a higher proliferative capacity. (**D**) Strain-stress curves for HaCGMs with HAp (0%, 1%, 3% and 5%) subjected to compression tests. (**E**–**F**) The porosity and swelling ratio of HaCGMs with HAp (0%, 3%). The data are the means ± SEM; n = 4.

HaCGMs with 3% HAp exhibited a better interconnected macroporous structure compared with HaCGMs with other HAp concentrations, as shown at different magnifications (Fig. 2A). The pore diameter distribution analysed with ImageJ and showed that pore diameters of HaCGMs with 0% HAp were in the range of 50–80 µm, HaCGMs with 1% or 3% HAp were mainly in the range of 30–50 µm, and the pores of HaCGMs with 5% HAp were in the range of 10–30 µm (Fig. 2B). These results indicated that the presence of HAp in the HaCGMs reduced pore sizes. Live/dead staining with Calcein AM/PI showed that IPFP-ASCs seeded in HaCGM with 3% HAp possessed better proliferative capacity after 3 days, while cells survived poorly in HaCGMs with 5% HAp, indicating that macroporous structures have a great influence on cells (Fig. 2C, Sup. Figure 1). These results indicated that introduction of HAp into micro-scaffolds at controlled concentrations did not affect the biocompatibility of the micro-scaffolds. The mechanical capacities of HaCGMs with different concentrations of HAp were compared using a compression test (Fig. 2D). Compared with pure gelatin micro-scaffolds, HaCGMs exhibited superior mechanical properties because they are stiffer. Despite the decrease in porosity and swelling ability, HaCGMs with 3% HAp still exhibited a relatively high porosity (>80%) and swelling ratio (>20) (Fig. 2E,F). Such decreases were within expectation because introduction of HAp decreased the micro-scaffolds pore size. Nonetheless, the HaCGMs with 3% HAp exhibited desirable material properties (including pore size, porosity and swelling ratios), excellent mechanical capacities and satisfactory cellular biocompatibility, and were thus chosen for following applications.

### Degradation of HaCGMs

**Figure 3:**
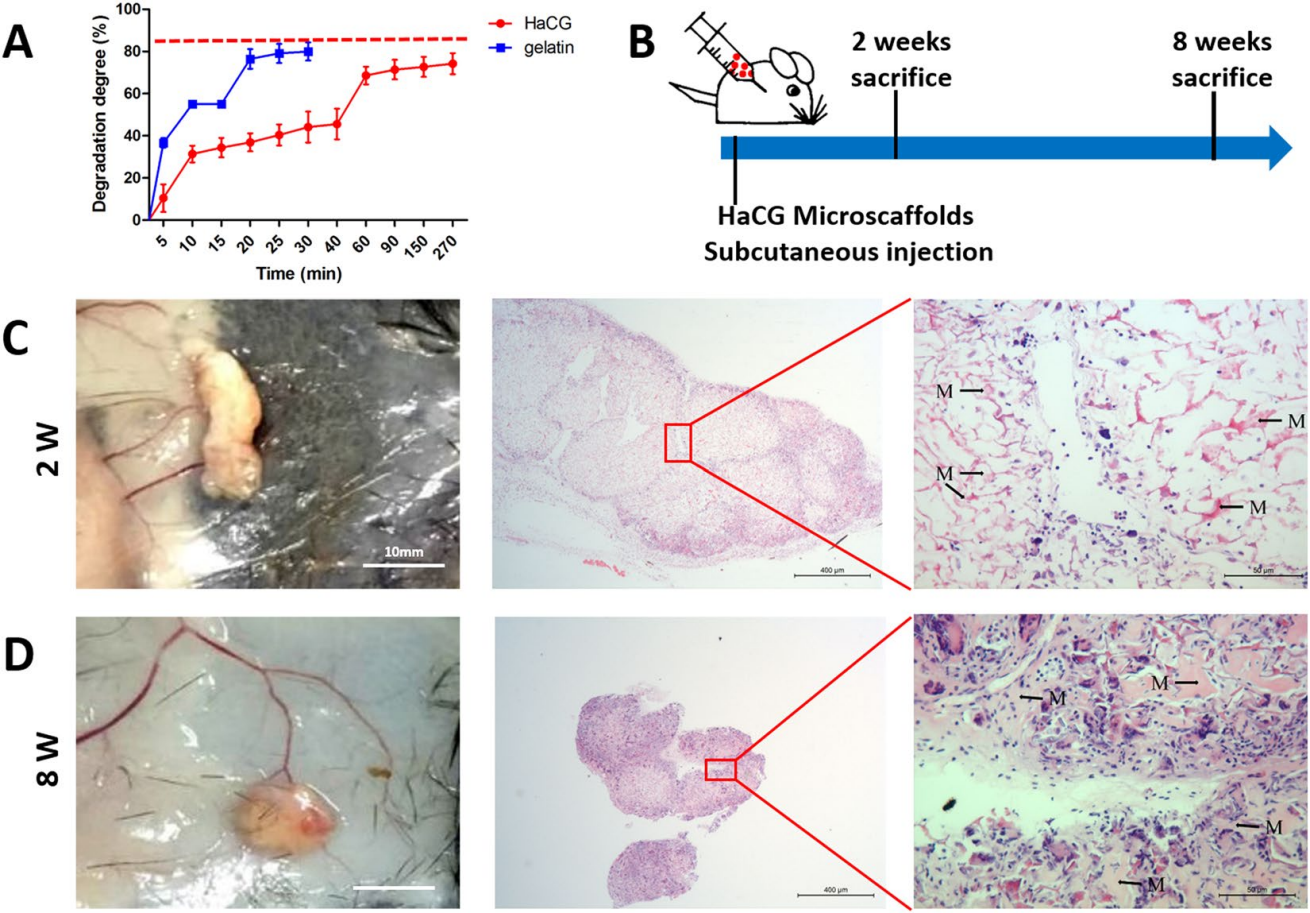
Degradation of HaCGMs. (**A**)Images showing the *in vitro* degradation rate of HaCGMs with 3% HAp and gelatin micro-scaffolds. (**B**) Schematic showing the *in vivo* degradation rate of HaCGMs with 3% HAp after subcutaneous injection. (**C**–**D**) Macroscopic view of HaCGMs after 2 weeks and 8 weeks. Note that dense and organized blood vessels were found to form around reddish fibrous capsules at the injection sites. H&E staining showing endogenous cell loading around or inside HaCGMs. M = Microscaffold. We got permission from Xiuxiu Wang for publication of the image.

Gelatin micro-scaffolds could be degraded within 30 min *in vitro* (~80% degradation) (Fig. 3A). However, the degree of HaCGMs degradation within 30 min was approximately 45%, and the degradation was 80% at 270 min, which indicated that the bioactivity and mechanical durability of HaCGMs can be maintained for a long time to support regeneration of subchondral bone (Fig. 3A). A similar observation was made *in vivo*. HaCGMs remained observable 2 weeks after subcutaneous injection, and took as long as 8 weeks to be significantly degraded. Dense and organized blood vessels were found to form around reddish fibrous capsules at the injection sites. H&E staining further showed endogenous cell loading around or inside HaCGMs, suggesting minimal induction of inflammation and a capacity for cellular infiltration (Fig. 3C,D).

### Characterization of infrapatellar-fat-pad-derived stem cells (IPFP-ASCs)

**Figure 4:**
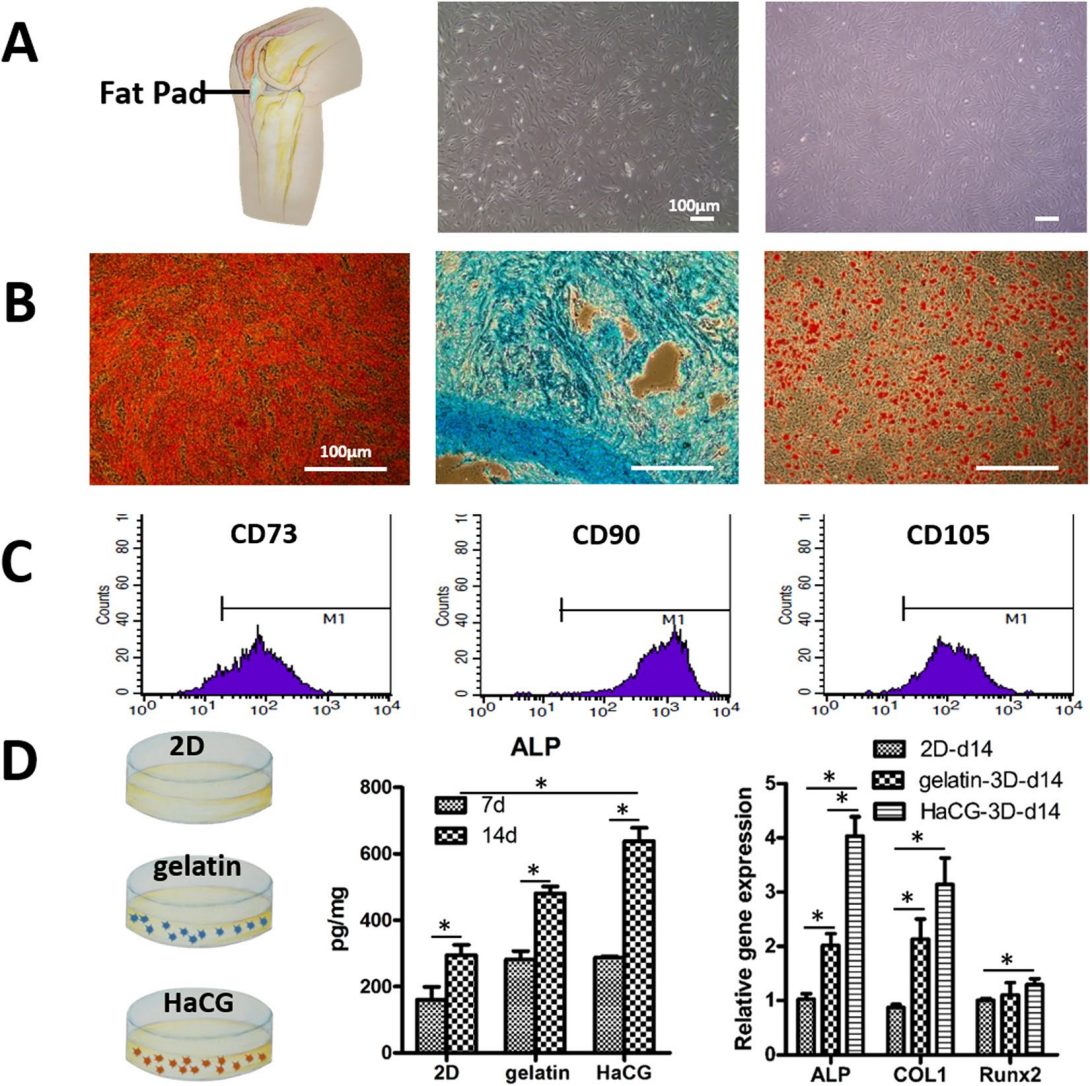
Characterization of infrapatellar-fat-pad-derived stem cells (IPFP-ASCs). (**A**) The location of the infrapatellar fat pad. IPFP-ASCs and bone marrow stem cells have flat polygonal morphology at the 3^rd^ passages under light microscope. (**B**) The multi-lineage differentiation ability of IPFP-ASCs was assessed by staining with appropriate specific reagents. Positive staining was observed following osteogenic, chondrogenic, or adipogenic differentiation using specific induction media. (**C**) Flow cytometry analysis of IPFP-ASCs revealed positive markers (CD 73, CD 90, CD 105). (**D**) IPFP-ASCs were encapsulated in HaCGMs and cultured in osteogenic induction medium, with gelatin microcryogels and 2-dimension (2D) culture as controls. ELISA analysis revealed higher ALP activity produced by cells cultured in HaCGMs on day 14 compared to day 7. Quantitative RT-PCR showed that the gene expression of osteoinductive markers, such as alkaline phosphatase (ALP), type I collagen (COL1), and RUNX2, were up-regulated. The data are expressed as the relative gene expression normalized to that of the housekeeping gene. The data are the means ± SEM; n = 4.

IPFP was weighed (approximately 10 g/knee) and cells were successfully isolated (approximately 5*10^6^/knee for primary culture). *In vitro* expansion of these cells from P0 to P3 took approximately 16 days. IPFP-ASCs of P3 had a flat polygonal morphology, which was similar to that of other types of mesenchymal stem cells (MSCs) (Fig. 4A). The multi-lineage differentiation ability of IPFP-ASCs was assessed by staining with appropriate specific reagents. Positive staining was observed following osteogenic, chondrogenic, or adipogenic differentiation using specific induction media respectively (Fig. 4B). Flow cytometry also revealed that IPFP-ASCs, such as other stem cells, express CD 73, CD 90, and CD 105 (Fig. 4C), but do not expressing CD 34 (Sup Fig. 2).

### Osteoinduction of IPFP-ASCs with HaCGMs

IPFP-ASCs were encapsulated in HaCGMs and cultured in osteogenic induction medium (Gibco, A10072), with cells encapsulated in gelatin micro-scaffolds and in 2D culture as controls. IPFP-ASCs proliferate well in HaCGMs after 14 days. Quantitative RT-PCR showed that genes expression of osteoinductive markers, such as alkaline phosphatase (ALP), type I collagen (COL1), and RUNX2, was up-regulated. ELISA analysis showed higher ALP activity produced by cells cultured in HaCGMs on day 14 compared to day 7, indicating the gradual maturation of IPFP-ASCs towards the osteogenic lineage (Fig. 4D).

### *In vivo* therapeutic effect of injectable HaCGM for subchondral bone lesions

**Figure 5:**
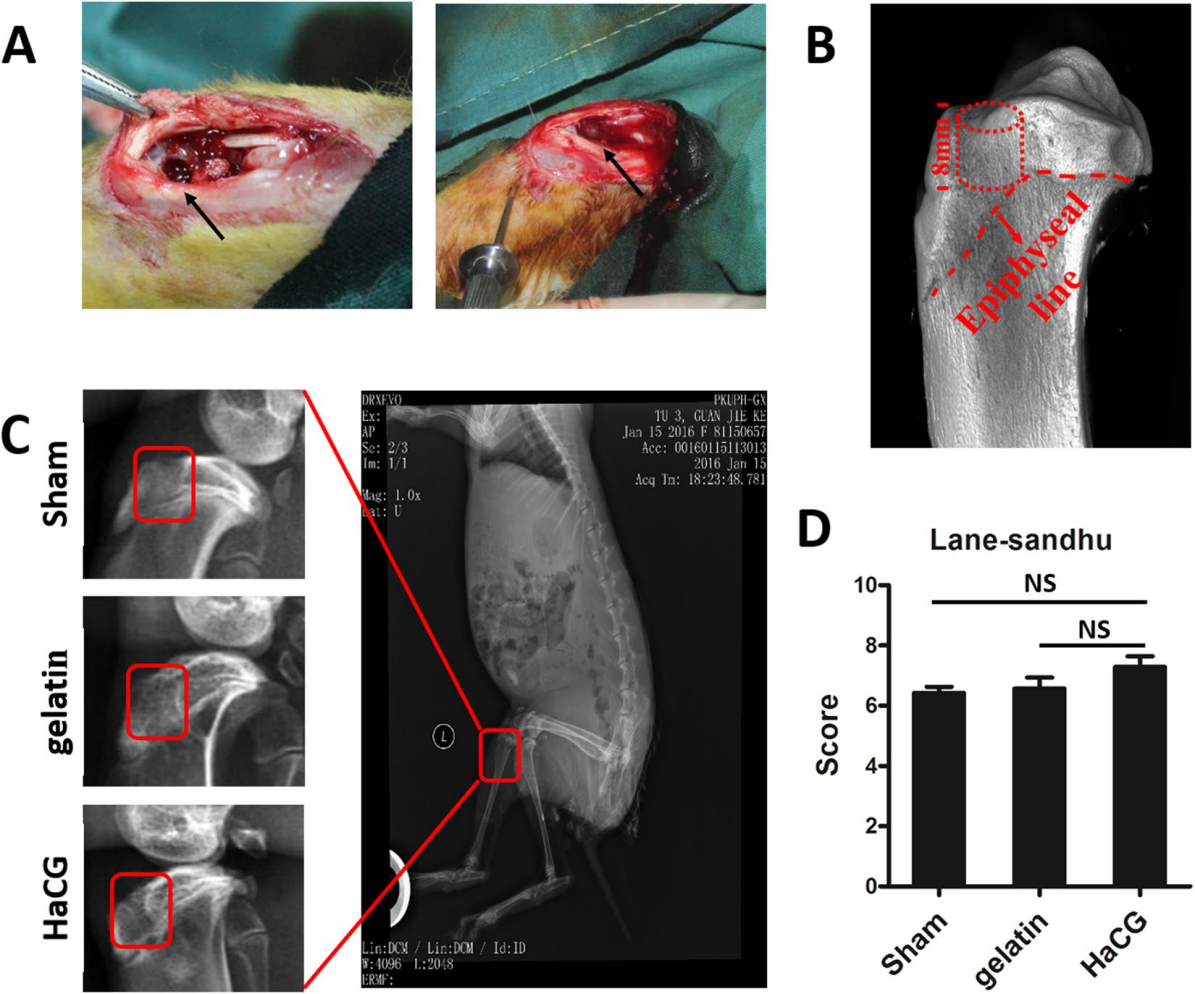
*In vivo* therapeutic effect of injectable HaCGMs for subchondral bone lesion. (**A**) HaCGMs were injected in a rabbit model of subchondral bone lesion, which imitated the actual necrosis removing of subchondral bone cysts. (**B**) Photographic images showing the details of subchondral bone lesions. (**C**) Full-body roentgenogram showing no fibrosis agent in the proximal metaphysis of the tibia or in the lungs at the same time. Lateral roentgenogram of the proximal tibia showed that rabbits treated with the HaCGMs had better regeneration. (**D**) Evaluation of the Lane-Sandhu radiological score. The data are shown as the means ± SEM; n = 6.

HaCGMs were injected into a rabbit model of sealed subchondral bone lesions, which imitated the necrosis removing of subchondral bone cysts before sequestrectomy (Fig. 5A,B). All the animals tolerated the bilateral model without complication and could bear the load of their own weight on the day after surgery. Full-body roentgenogram revealed no fibrosis or thrombosis in the proximal metaphysis of the tibia (Fig. 5C). No fibrosis or thrombosis was observed in the lung either, which had been reported to be an adverse indication in other research^30^. After 8 weeks, lateral roentgenograms of the proximal tibia showed that rabbits treated with HaCGMs scored better in the Lane-Sandhu assessment, though no significant difference was observed by X-ray due to resolution limitation (Fig. 5D). More sensitive instruments could potentially identify subtle differences.

### Assessment of subchondral bone lesions regeneration using micro-CT analysis

**Figure 6:**
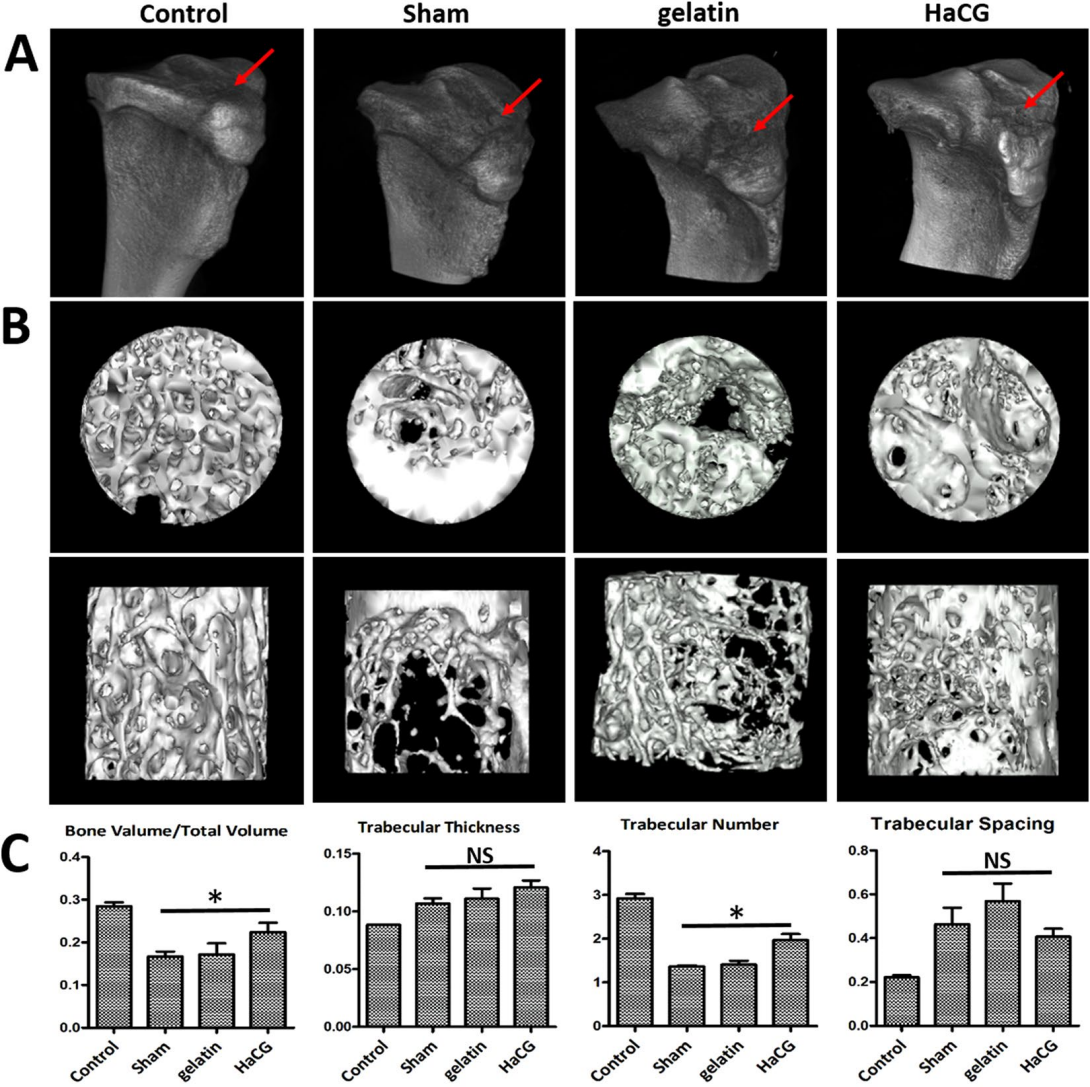
Assessment of subchondral bone lesion regeneration by micro-CT analysis. (**A**) The tibias surface of rabbits was evaluated by 3D reconstruction based on micro-CT images. (**B**–**C**) micro-CT was used to generated 3D reconstruction of subchondral lesion that showed regeneration differences among the different groups. (**D**) The parameters of microarchitecture (BV/TV, Tb. N, Tb. T, and Tb. S) were measured in the trabecular bone of the proximal tibia (1–2 mm distal to the proximal physis). HaCGM treatment induced a significant increase in BV/TV and Tb. N compared with other treatments (*p* < 0.05). No difference was observed in Tb. T and Tb. S. BV/TV = bone volume/total volume; Tb. N = trabecular number; Tb. T = trabecular thickness; Tb. S = trabecular spacing. The data are shown as the means ± SEM; n = 6.

Micro-CT was then used to assess the macrostructure and microarchitecture of bone, bone mineral density (BMD) and 3-dimensional (3D) bone morphometry for a better comparison. Animals treated with HaCGMs possessed better tibia joint flatness compared to other treatment groups, as visualized by 3D reconstruction of micro-CT images (Fig. 6A). Accurate locations of bone generation from transverse, coronary, and sagittal directions in the ROI are shown (Fig. 6B). 3D reconstruction of subchondral bone lesions showed regeneration differences among the different groups. The parameters of microarchitecture (BV/TV, Tb. N) were measured in the trabecular bone of the proximal tibia (1–2 mm distal to the proximal physis). HaCGMs treatment induced a significant increase in BV/TV and Tb. N compared with the other treatments (*p* < 0.05). No difference was observed in Tb. T and Tb. S (Fig. 6C).

### Histopathological examination

**Figure 7:**
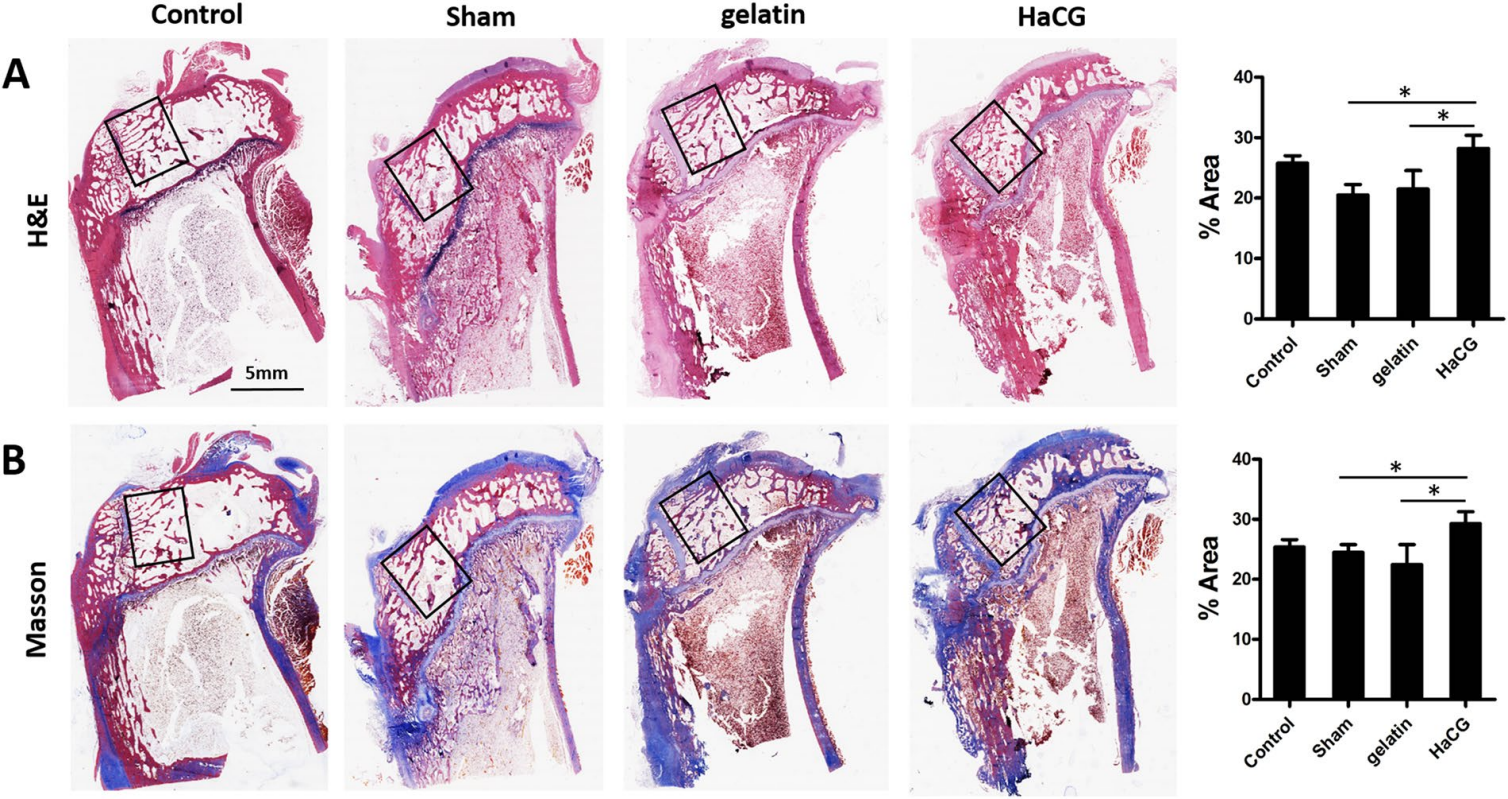
Histopathological analysis of subchondral bone regeneration. (**A**–**B**) Representative histological H&E and Masson trichrome staining of longitudinal sections of tibia at 8 weeks post-surgery. Diagram showing the relative trabecular area. Data are shown as the means ± SEM; n = 9.

**Figure 8:**
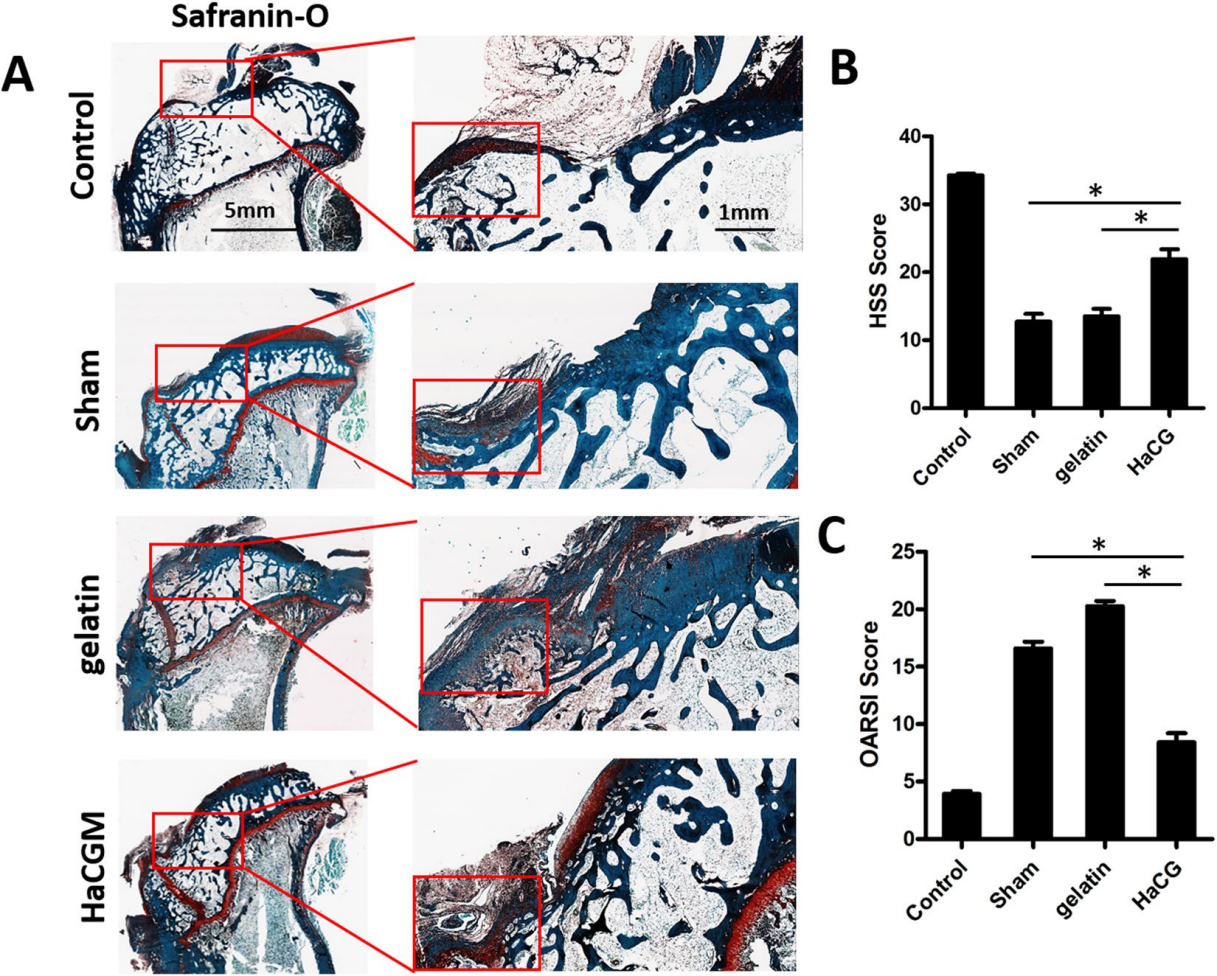
Histological analysis of the protective role of subchondral bone regeneration with regard to the upper cartilage. (**A**) Representative histological safranin-O & fast green staining of longitudinal sections of tibia at different magnifications. (**B**) Diagram showing the results of histological grading evaluated at 8 weeks post-surgery according to OARSI scores and HSS scores. The data are shown as the means ± SEM; n = 9.

Eight weeks post-surgery, H&E (Fig. 7A) and Masson trichrome staining (Fig. 7B) were performed on longitudinal sections from the proximal metaphysis of the tibia. Animals treated with HaCGMs generated tissues that were more similar to normal subchondral bone, hence exhibiting the most-satisfying bone regeneration results compared with the other treatment groups. These results were further confirmed by analysis of the relative trabecular area, in which animals treated with HaCGMs showed more trabecular regeneration (Sup. Figure 5). Moreover, safranin O-fast green staining (Fig. 8A) revealed that HaCGMs treatment helped better preserve the integrity of the cartilage above the subchondral bone lesions. Using the Histological Scoring System (HSS) and Osteoarthritis Research Society International (OARSI) histological grading system to measure the degree and quality of cartilage reservation and subchondral bone regeneration, we found that animals treated with HaCGMs received relatively different scores from animals in other treatment groups (Fig. 8B). Together, these finding suggested that HaCGMs could effectively promote filling of subchondral bone lesions, preserve cartilage and facilitate subchondral bone regeneration.

## Discussion

Subchondral bone has been identified as an attractive target for KOA treatment. However, no effective treatment for existing inactivated subchondral bone lesions (such as subchondral bone cysts) accompanied by KOA had been established. In the present study, an injectable and biocompatible HaCGMs, with osteoinductive capability was proposed as a minimally invasive treatment for sealed subchondral bone lesions model. We established a sealed subchondral bone lesion model to mimic the actual necrosis removing performed by surgeons before sequestrectomy, and evaluated the therapeutic effects of the HaCGMs on subchondral bone regeneration *in vivo*. Using 3D reconstruction based on micro-CT scans and safranin O-fast green staining of the tibia joint, HaCGMs were shown to exert a protective effect and preserve the upper cartilage in the process of subchondral bone regeneration. Using these bio-functional HaCGMs, limitations of subchondral bone cysts treatment in the clinic may be partially overcome and HaCGMs could possibly lead to a promising approach for KOA treatment through resolution of subchondral bone lesions (such as subchondral bone cysts).

In our composite biomaterial, we combined chitosan and HAp, which have been shown to be biocompatible and bioactive when used for bone regeneration^24^. The presence of HAp could further complement the artificial pro-osteogenic niche in our 3D porous micros-caffolds by enhancing osteoinduction. We found that different concentrations of HAp could alter the micro-scaffold morphology, absorbability, and stiffness, which is consistent with finding in other studies^25^. These different stiffnesses may lead to different osteoinduction properties^31,32^. Apart from the chemical composition of the material, the geometry and macrostructural properties have been shown to play a role in osteoinduction^33^. In addition, microstructural surface properties, including grain size, microporosity, surface roughness and specific surface area have been found to play a great role in osteoinduction^33^. Moreover, the multi-pore structures of micro-scaffolds is considered to be a key factor in directing cell-cell interactions during mesenchymal condensation; and permits exchange of growth factors, nutrients and waste^34–36^.

Thus, in our work, we successfully validated the biocompatibility and osteoinductivity of HaCGMs *in vitro* using human infrapatellar-fat-pad-derived stem cells (IPFP-ASCs). These stem cells are feasibly obtained from surgical samples, and maintained potency towards multi-lineage differentiations. Hence, IPFP-ASCs could possibly be used as an autologous cell source for treating of KOA^37^. Compared with other stem cells from fat, we found that the IPFP-ASCs had advantages, such as easy obtainment via either open or arthroscopic resection, comparable proliferation, superior osteogenic and chondrogenic differentiation. We have built our IPFP-ASCs sourcing, culturing, and identifying system, and we just used this type of ASCs as a model cell population in this research. In addition, because we observed host cell infiltration into HaCGMs *in vivo*, it is possible that in our animal model, the ingrown cells and signaling elements were provided by the marrow of the proximal tibial, and combining these with essential bone-growth cytokines from the HaCGMs, promoted regeneration of the subchondral bone lesions. Hence, we also hold high hopes for HaCGMs to recruit MSCs (such as bone marrow stem cells or tumour stem cells) to damaged sites and induce them to an osteogenic lineage for *in situ* subchondral bone regeneration.

Despite the above strengths of HaCGMs demonstrated in this study, several limitations exist. We did not observe osteoinductivity of HaCGMs without the use of an osteogenic induction medium, which was different from observations made by other researchers using nanohydroxyapatite/chitosan/gelatin materials^38^. This indicates that the current HaCGMs still needs to be optimized, for example, by conjugating BMP-2 to the HaCGMs^39^, for enhanced osteoinduction. Clinical KOA progression is known to be chronic, requiring repeated stimulations or injury. It is difficult to propose ideal subchondral bone cyst in animal knees, therefore we performed one large subchondral cavity to mimic the actual necrotic region removing performed by surgeons before sequestrectomy. The regenerative progression after sequestrectomy and injection was similar to the clinical treatment of femoral head necrosis^40^. Thus, this animal model, developed to mimic the reparative progression of subchondral lesions was acceptable; but still needs to be studied in the future. Additionally, the ability of rabbits to heal spontaneously needs to be considered. Despite achieving relatively good osteoinduction in subchondral bone and preserving cartilage using HaCGMs, the mechanism behind the effect of subchondral bone regeneration on cartilage preservation remains unclear. There has been some speculations that molecular crosstalk exists between the cartilage and subchondral bone due to infiltration of catabolic agents via small channels and blood vessels^41,42^. Studies have shown that cytokines secreted by subchondral bone can alter cartilage metabolism^43^. Such metabolism may play a role in our study. We investigated only the effect of HaCGMs on IPFP-ASCs *in vitro*, and future studies are still required to verify whether HaCGMs loaded with autologous cells would be a better alternative for knee subchondral lesion regeneration.

In conclusion, the novel HaCGMs synthesized in this study shows great promise as a potential niche for subchondral bone regeneration *in vitro* and *in vivo* without the use of exogenous cells. This surgical strategy with a one-step injection provides a new approach for regeneration of subchondral bone lesion; and may represent an attractive modification strategy for treating KOA.

## Methods

### HaCGMs fabrication

HaCGMs were fabricated as previously described^34^. Briefly, cylindrical micro-scaffolds with diameters of 400 µm and heights of 600 µm were fabricated. HaCG solutions prepared in water with 2% gelatin, 1.25% chitosan, and varying hydroxyapatite percentage (HAp) (0%, 1%, 3% and 5%) were manually scraped onto a microstencil array chip and cryogelated at −20 °C for 20 h to form microscaffold array chips. Microscaffold array chips were then lyophilized for 2 h; and harvested by a simple push-out method using a PDMS ejector pin array to obtain micro-scaffolds. Harvested micro-scaffolds were collected by filtering through a 70-µm cell strainer and resuspended at the desired densities in 1% carboxymethyl cellulose (CMC) for homogeneous distribution. Harvested HaCGMs were freeze-dried and sterilized using ethylene oxide.

### Scanning electron microscopy (SEM) of the HaCGMs

HaCGMs were imaged with SEM to evaluate their microstructures. Freeze-dried HaCGMs were gold-coated for 90 s prior to SEM (FEI Quanta 200, USA). HaCGMs pore size distribution was measured and analysed in 10 SEM images from seven different areas using ImageJ software (National Institutes of Health).

### Compression, swelling ratio and porosity of HaCGMs

Compression tests were performed using a Bose 3230 mechanical testing instrument (Bose, USA). HaCGMs with varying concentrations of HAp (each with four parallel samples) were tested, and average values were used to plot stress-strain curves. The swelling ratio of HaCGMs was determined as the ratio of the weight of swollen HaCGMs to that of dried HaCGMs. To evaluate porosity, dried HaCGMs were hydrated sufficiently and weighed (w1). Retained water was carefully blotted up and HaCGMs were then weighed again (w2). Finally, porosity was calculated as porosity = (w1-w2)/w1.

### Degradation of HaCGMs *in vitro* and *in vivo*

To demonstrate the biodegradability of HaCGMs with 3% HAp and gelatin micro-scaffolds *in vitro*, harvested and freeze-dried micro-scaffolds were immersed in 0.025% commercial trypsin/EDTA solution (Gibco). At predefined time points (t = 10, 15, 20, 25, 30, 40, 60, 90, 150, and 270 min), the loss in dry weight was measured and calculated based on the formula for degradation testing^44^. For *in vivo* assessment, approximately 400 micro-scaffolds suspended in 1 ml of PBS solution were injected subcutaneously into C57/6 L mice (20–25 g, 4–6 weeks). The mice were sacrificed after 2 weeks and 8 weeks, and implants were detached for macroscopic viewing and histological staining.

### Isolation and culture of infrapatellar-fat-pad-derived adipose stem cells (IPFP-ASCs)

Human infrapatellar-fat-pad-derived adipose stem cells (IPFP-ASCs) were sourced from patients undergoing knee arthroplasty surgery at the Peking University People’s Hospital, Beijing, P.R. China. The study was approved by the Peking University Health Science Center Ethics Committee, and informed consent was obtained from all patients. We confirmed that all experiments were performed in accordance with relevant guideline and regulations. To isolate IPFP-ASCs, approximately 10 g of infrapatellar fat pad was washed 3–4 times with HBSS solution to remove blood thoroughly and then cut into small pieces. The tissue was digested in 10 ml 0.1% w/v collagenase type I (Gibco) for 6 h at 37 °C. IPFP-ASCs were then suspended in DMEM (Gibco) containing 10% foetal calf serum (FCS)_._ The medium was changed on day three to remove non-adherent cells and blood cells. Cells of passage 3–5 (P3-P5) were used for the following experiments. Positive cell surface markers (CD 73, CD 90, CD 105) and negative cell surface markers (CD34, CD31) were evaluated with flow cytometry. Osteogenic, chondrogenic, and adipogenic differentiation potential were determined by culturing in specific medium and staining with Alizarin Red, Alcian Blue, and Oil Red, respectively. The pellet 3D culturing of IPFP-ASCs was used to identify the chondrogenic potential.

### Cell encapsulation and culture in gelatin micro-scaffolds and HaCGMs

IPFP-ASCs were suspended at a final concentration of 1×10^7^ cells/ml. Then, 200 µl of cell suspension was slowly and evenly pipetted onto dried and sterile gelatin micro-scaffolds and HaCGMs. After 2 h of incubation to allow IPFP-ASCs to absorb and attach in the micro-scaffolds, 3 ml of osteogenic induction medium (Gibco, A10072) was slowly added to submerge all the micro-scaffolds. Cells in the micro-scaffolds were then cultured at 37 °C, 5% CO2 for 7–14 days. The medium was exchanged with fresh medium every 2–3 days. Cell viability was assessed with Calcein-AM and PI staining, and stained cell were observed with a fluorescence microscope (Nikon).

### RT-PCR

Total RNA was extracted using Trizol (Invitrogen) according to the manufacturer’s protocol. Reverse-transcription was carried out using ReverTra Ace Qrna RT Master Mix with gDNA Remover (TOYOBO). Samples were denatured for 30 sec at 95 °C and then amplified for 40 cycles as follows: denaturation at 95 °C for 5 sec, annealing at 55 °C for 10 sec and extension at 72 °C for 15 sec. The Ct values of the products were normalized to that of the internal control, glyceraldehyde-3-phosphate dehydrogenase (GAPDH), and expression levels of genes of interest were calculated with the 2-△△CT method. The primer details are shown in Table 1.

**Table 1:** Primer sequences.

Primer	Sequences
Collagen I	
Sense	GCGAAGGCAACAGTCGCT
Antisense	CTTGGTGGTTTTGTATTCGATGAC
ALP	
Sense	ACAAGCACTCCCACTTCATC
Antisense	ATTCTGCCTCCTTCCACC
Runx2	
Sense	GTGGACGAGGCAAGAGTT
Antisense	GGTGCAGAGTTCAGGGAG
GAPDH	
Sense	GAAGGTCGGAGTCAACGG
Antisense	GGAAGATGGTGATGGGATT

### ALP activity assay

An ALP activity assay was performed to evaluate the osteoinductive effect of HaCGMs on IPFP-ASCs. Cells were incubated at 37 °C, 5% CO2 for 7 and 14 days, and the medium was changed twice a week. During medium changes, the cell culture supernatant was collected and stored at −80 °C for further assay. For ALP assays, 50 µl of supernatant was mixed with 150 µl of ALP assay reagent according to the manufacturer’s protocol (Multi Sciences, Beijing). The final ALP activity of cells on day 7 was calculated as the sum of ALP activity on day 3 and day 7. The final ALP activity of cells on day 14 was the sum of that on day 3, day 7, day 10 and day 14.

### Animal model

All animal experiments were conducted in accordance with the guidelines for the care and use of laboratory animals and were approved by the Animal Care and Use Committee of Peking University People’s Hospital. In brief, 24 adult male New Zealand white rabbits (2–2.5 kg) were purchased from the Laboratory Animal Center of Peking University People’s Hospital. The animals received treatments randomly according to the following groups in this study: Sham, gelatin, and HaCGM. Each group included 8 animals. Surgery was performed under general anesthesia via weight-adapted intravenous injection of 4% pentobarbital (1 ml/kg body weight) in a hospital laboratory. Both hind limbs of each animal were shaved, and the skin was sterilized with iodine and ethanol. The tibial tubercle was palpated and identified. An osteochondral plug was removed with a hand-driven corneal trephine, and a 4-mm hole was drilled vertically through the tibial eminence to the epiphyseal line at the proximal metaphysis. The cavity was then bluntly reamed with a steel wire (4 mm diameter), and the depth of the cavity was approximately 8 mm, which did not pass the epiphyseal line. The width and depth of the medullary cavity were carefully monitored. The entire osteochondral plug was put back in the hole to make a cavity prior to scaffold injection. A paracentetic needle was inserted into the cavity from the lateral side of the proximal tibial metaphysis until there was a sense of breakthrough. HaCGMs and gelatin micros-caffolds that had been prepared in advance were injected. No material leakage was observed. The procedures were carried out at three different afternoon. Gentamicin was used postoperatively, and animals were kept in separate cages after surgery in a pathogen-free facility with 12-h light, 12-h dark cycle; and fed a normal diet.

### Radiographic and micro-CT analysis

Lateral-view and full-body radiographs were obtained at week 4 and week 8 after surgery. Recovery of new bone in the cavity was observed and analysed according to Lane-Sandhu score by two independent investigators^45^. The score details are shown in Sup. Table 2. Images were acquired at an effective pixel size of 13.57 μm, voltage of 80 kV, current of 500 μA and exposure time of 1,500 ms in each of the 360 rotational steps. The reconstructed data of cylindrical regions of interest (ROIs), with a diameter of 4 mm and height of 6 mm were examined using Inveon Research Workplace (Siemens) software. The parameters used for calculating the trabecular bone of ROI were as follows: bone volume/total volume (BV/TV), bone surface area/bone volume (BS/BV), trabecular thickness (Tb.Th), trabecular number (Tb.N), and trabecular separation (Tb.Sp) according to guidelines set by the American Society for Bone and Mineral Research^46^.

### Histological analysis

Tibiae were fixed in 10% formalin in phosphate-buffered saline for a minimum of 72 h prior to decalcification with EDTA for 3 weeks. After a series of dehydrations and fixations, samples were embedded in paraffin. Tibiae were cut longitudinally (5 μm) along the anteromedial cortex using the marker pins placed to identify the central portion of the lesion. HE and Masson’s trichrome staining were used to detect bone ingrowth, remaining implant material and soft tissue voids at each time point under light microscopy. The relative trabecular area was analysed with ImageJ software. The Osteoarthritis Research Society International (OARSI)^47^ score and Histological Scoring System (HSS)^48^ were used to grade cartilage reservation after regeneration of subchondral lesion after safranin O and fast green staining. The grading score details are shown in Sup. Tables 3 and 4.

### Statistical analysis

All analyses were conducted using SPSS (version no.20 and IBM SPSS Statistics). All data are presented as the mean ± SEM. To test the significance among groups, one-way ANOVA analysis of variance was applied. A value of *p* < 0.05 was considered statistically significant.

## Electronic supplementary material

Supplementary Information

The procedure of surgical
