# Supplementary material for: Injectable nanohydroxyapatite-chitosan-gelatin micro-scaffolds induce regeneration of knee subchondral bone lesions
Source: Sci Rep. 2017 Dec 1;7:16709. doi: 10.1038/s41598-017-17025-6 (PMC5711958; doi:10.1038/s41598-017-17025-6)
Supplement: Supplementary file 1 — Supplementary Information [file 41598_2017_17025_MOESM1_ESM.pdf]

Injectable nanohydroxyapatite-chitosan-gelatin microscaffolds induce regeneration of knee subchondral bone lesions

B. Wang [a,c *], W. Liu [b, *], D. Xing [a], R. Li [a], C. Lv [b], Y. Li [b], X. Yan [b], Y. Ke [a], Y. Xu [a], Y. Du [b, ##], J. Lin [a, #]

Sup Fig.1. Schematic showing the quantification data of cell proliferation, which shown that HaCGM possessed a better proliferative capacity.

Sup Fig.2. Schematic showing the identified marker of IPFP-ASCs by flow cytometry.

Sup Fig.3. Schematic illustration of surgical procedures. All aseptic surgical procedures were completed in standard sterile operating room.

Sup Fig.4. Schematic showing the accuracy of location of bone generation from transverse, coronary, and sagittal directions of ROI. ROI= regions of interest.

Sup Fig.5. Schematic showing the higher magnification images of ROI from Figure 7 and the relative area of trabecular.

Sup Fig.1.

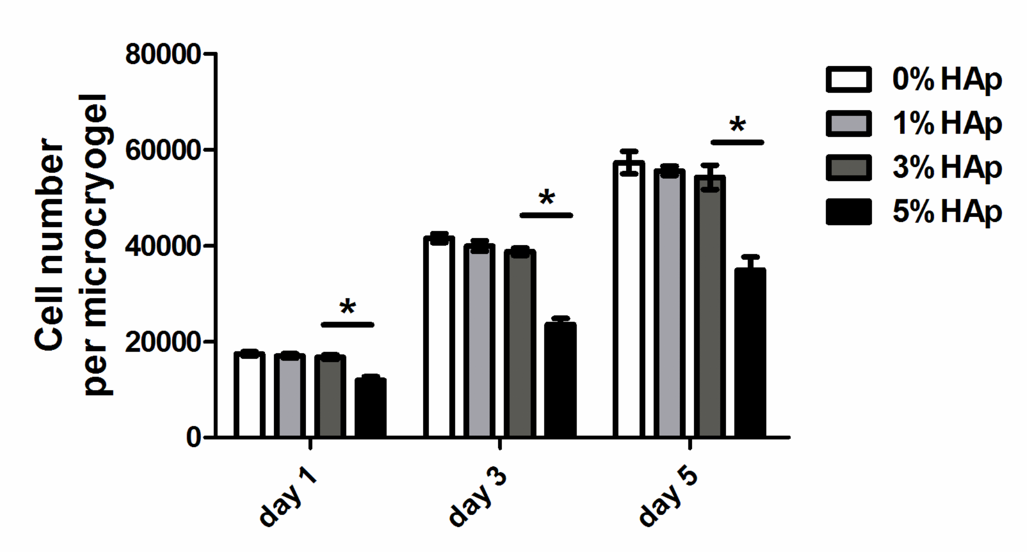

Sup Fig.2.

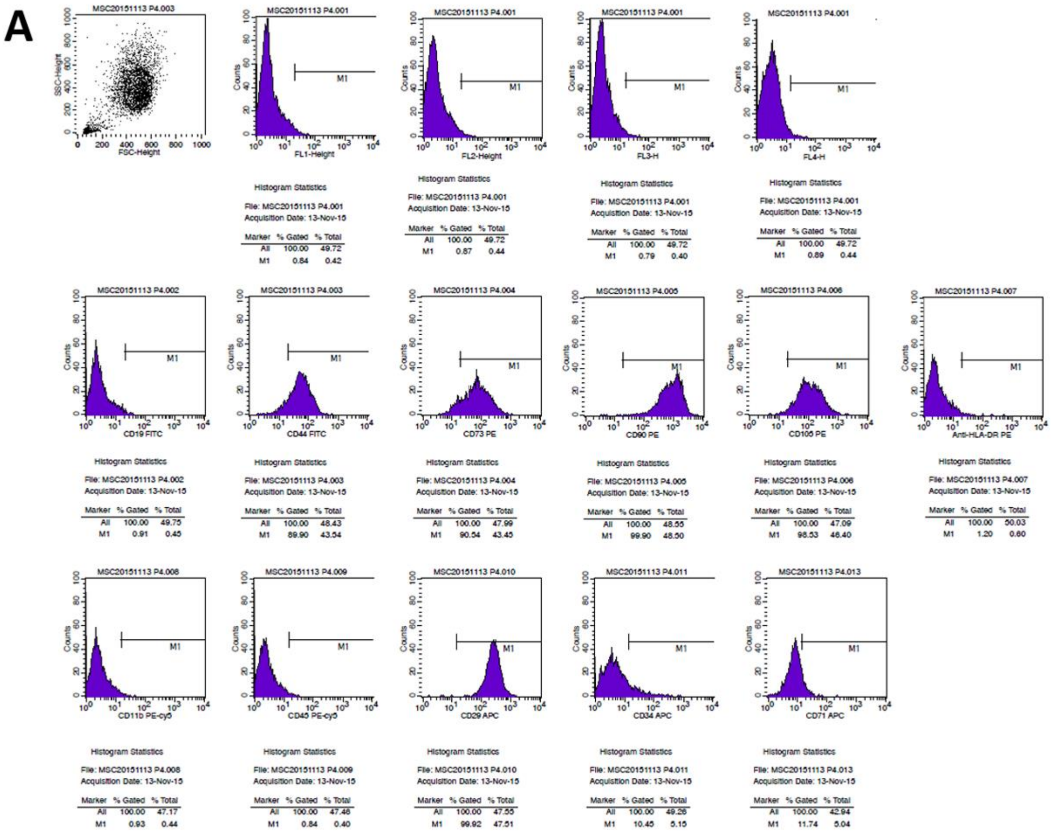

Sup Fig.3.

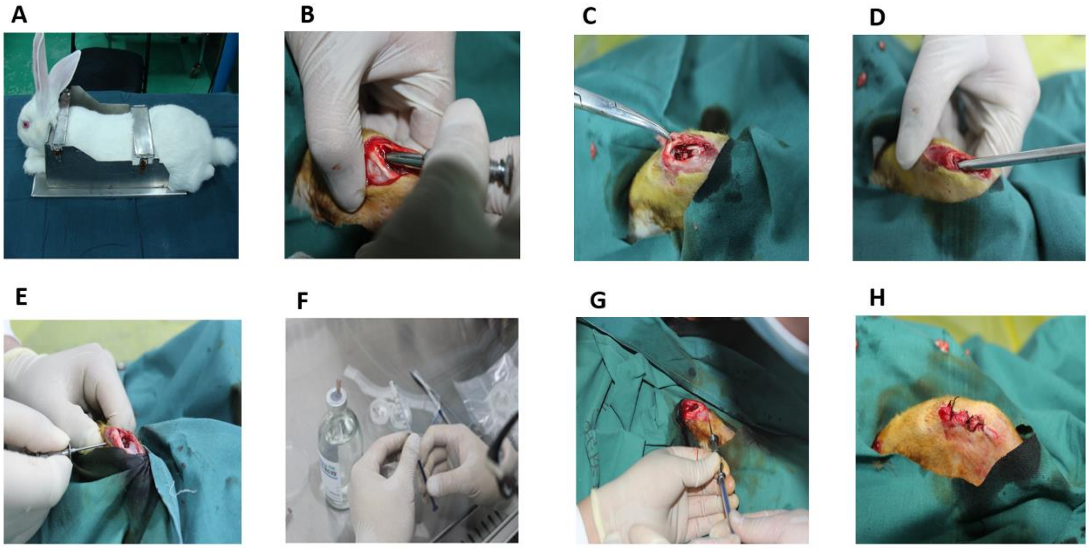

Sup Fig.4.

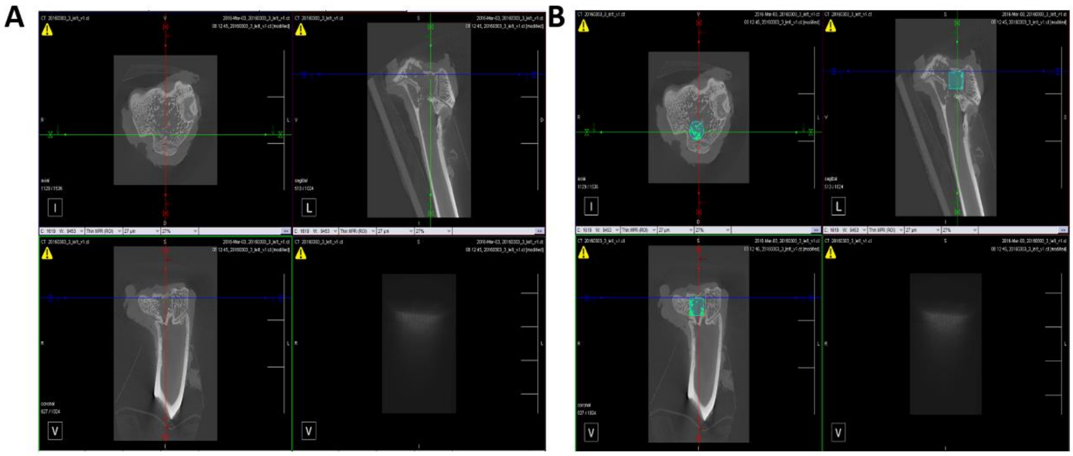

Sup Fig.5.

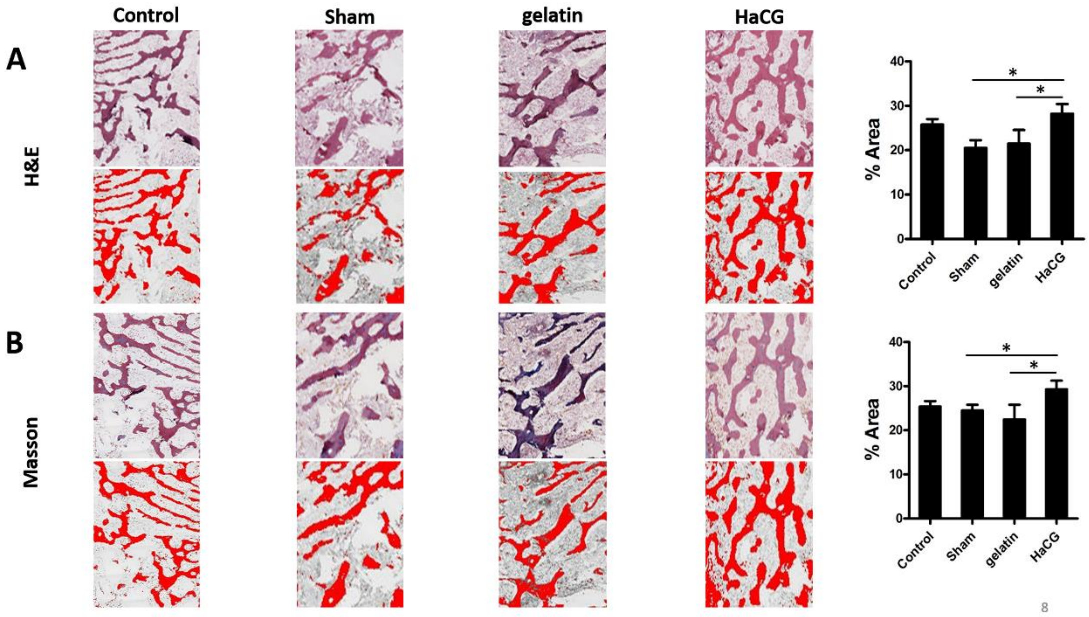

Control
Sham
gelatin
HaCG
A
H&E
B
Masson
% Area
40
30
20
10
0
*
*
Control
Sham
gelatin
HaCG

Sup Table 1: Description of Lane-Sandhu radiographic scoring

Lane-Sandhu	
Score	Description
0	No callus
1	Minimal callus formation
2	Callus evident and beginning osseous formation
3	Callus evident and fracture line almost obliterated
4	Complete union with complete remodelling

Sup Table 2: OARSI Score

**Parameter**
**Safranin O-fast green staining**
0=uniform staining throughout articular cartilage
1=loss of staining in superficial zone of hyaline cartilage<50% the length of the condyle or plateau
2=loss of staining in superficial zone of hyaline cartilage≥50% the length of the condyle or plateau
3=loss of staining in the upper 2/3’s of hyaline cartilage<50% the length of the condyle or plateau
4=loss of staining in the upper 2/3’s of hyaline cartilage≥50% the length of the condyle or plateau
5=loss of staining in all the hyaline cartilage<50% the length of the condyle or plateau
6=loss of staining in all the hyaline cartilage≥50% the length of the condyle or plateau
**Structure**
0=normal
1=surface irregularities
2=fissures in <50% surface
3= fissures in ≥50% surface
4=erosion 1/3 hyaline cartilage <50% surface
5=erosion 1/3 hyaline cartilage ≥50% surface
6=erosion 2/3 hyaline cartilage <50% surface
7=erosion 2/3 hyaline cartilage ≥50% surface

8=Full depth erosion hyaline cartilage<50% surface

9=Full depth erosion hyaline cartilage ≥50% surface

10=Full depth erosion hyaline and calcified cartilage to the subchondral bone <50% surface

11=Full depth erosion hyaline and calcified cartilage to the subchondral bone ≥50% surface

Chondrocyte density

0=no decrease in cells

1=focal decrease in cells

2=multifocal decrease in cells

3=multifocal confluent decrease in cells

4=Diffuse decrease in cells

Sup Table 3. Histological scoring system for rabbit osteochondral lesions.

Histological scoring system for evaluation in rabbit osteochondral lesions.		Score
**(a)**	**Over all defect evaluation (Throughout the entire defect depth)**	
1.	Percent filling with newly formed tissue	
	100%	3
	>50%	2
	<50%	1
	0%	0
2.	Percent degradation of the implant	
	100%	3
	>50%	2
	<50%	1
	0%	0
**(b)**	**Subchondral bone evaluation(within the subchondral bone area)**	
3.	Percent filling with newly formed tissue	
	100%	3
	>50%	2
	<50%	1
	0%	0
4.	Subchondral bone morphology	
	Normal,trabecular bone	4
	Trabecular bone, both some compact bone	3
	Compact bone and fibrous tissue	2
	Only fibrous tissue or no tissue	1
5.	Extent of new tissue bonding with adjacent bone	
	Complete on both edges	3
	Complete on one edge	2
	Partial on both edges	1
	Without continuity on either edge	0
**(c)**	**Cartilage evaluation (within the cartilage area)**	
6.	Morphology of newly formed surface tissue	
	Exclusively articular cartilage	4
	Mainly hyaline cartilage	3
	Fibrocartilage(spherical morphology observed with ≥75% of cells)	2

	Only fibrous tissue(spherical morphology observed with < 75% of cells)	1
	No tissue	0
7.	Thickness of newly formed cartilage	
	Similar to the surrounding cartilage	3
	Greater than the surrounding cartilage	2
	Less than the surrounding cartilage	1
	No cartilage	0
8.	Joint surface regularity	
	Smooth, intact surface	3
	Surface fissures(<25% of new sureface thickness)	2
	Deep fissures(≥25% of new surface thickness)	1
	Complete disruption of the new surface	0
9.	Chondrocyte clustering	
	None at all	
	<25% chondrocytes	
	25-100% chondrocytes	
	No chondrocytes present(no cartilage)	
10	Chondrocyte and GAG content of new cartilage	
	Normal cellularity with normal Safranin O staining	3
	Normal cellularity with moderate Safranin O staining	2
	Clearly less cells with poor Safranin O statning	1
	Few cells with no or little Safranin O staining or no cartilage	0
11	Chondrocyte and GAG content of adjacent cartilage	
	Normal cellularity with normal Safranin O staining	3
	Normal cellularity with moderate Safranin O staining	2
	Clearly less cells with poor Safranin O staining	1
	Few cells with no or little Safranin O staining or no cartilage	0
